# A novel reactive-extractive distillation process for separation of water/methanol/tetrahydrofuran mixtures

**DOI:** 10.1038/s41598-024-52427-3

**Published:** 2024-01-22

**Authors:** F. Neyestani, R. Eslamloueyan

**Affiliations:** Chemical Engineering Dept., School of Chem. and Petrol. Engineering, Shiraz University, Shiraz, Iran

**Keywords:** Engineering, Chemical engineering

## Abstract

The design of separation systems for the purification of azeotropic mixtures is of great importance in the chemical industries from economic and environmental points of view. Two novel reactive-extractive distillation (RED) processes, new design (I) and new design (II), were proposed for separating the azeotropic mixture of water/methanol/tetrahydrofuran (THF). These processes were compared to a conventional extractive distillation (ED) process. New design (I) employs dimethyl sulfoxide as a solvent, while new design (II) utilizes ethylene glycol. Ethylene oxide was introduced to the first column in both designs, enabling the in-situ production of ethylene glycol, a valuable byproduct. This is a novel solution to separate water from the azeotropic mixture by reaction between ethylene oxide and water. Aspen Plus software was used to simulate and design the processes. Both suggested designs were compared economically with the base design which is an ED process. According to the results, the new design (I) is more cost-effective and environmentally friendly alternative to the base design and the new design (II). It has a lower total capital cost and produces less carbon dioxide. Additionally, it generates a valuable by-product, ethylene glycol, which can be sold for substantial revenue. As a result, the new design (I) is the preferred choice for replacing the conventional extractive distillation method.

## Introduction

In today's competitive market, manufacturing industries are under pressure to produce products at the lowest possible cost. The oil, gas, and petrochemical industries are particularly sensitive to cost, as their products are commodities that are traded on global markets. These industries are constantly looking for ways to reduce their costs, such as by optimizing their production processes, using cheaper raw materials, and reducing energy consumption. Any improvement in efficiency or cost reduction can lead to significant profits for these industries. Manufactured products should be designed and produced in such a way that their environmental impact is minimized while still achieving the essential requirements and price point^1^. Environmental pollution by pollutants such as nitrogen oxides (NOX), sulfur oxides (SOX), dust, and greenhouse gases released from industries has been given much attention by environmental organizations. In this field, the Paris Agreement, Montreal, and Kyoto Protocols have been ratified to tackle the climate change problem^2^.

Process industries require fossil fuels to meet their energy needs, which include a significant amount of steam and electricity. As a result, they release pollutants into the atmosphere, mostly carbon dioxide, a greenhouse gas. Therefore, these industries are trying to reduce the amount of carbon dioxide production by considering measures to adapt themselves to international environmental regulations. Hence, the process engineers seek to optimize the energy consumption of the processing plants and to design new processes having less energy consumption and GHG production^3^. Distillation units are one of the energy-intensive systems in the chemical and petrochemical industries, so their optimal design has a significant effect on reducing energy consumption and greenhouse gas production in the entire plant^4,5^.

In this study, we have worked on the separation of the ternary azeotropic mixture of methanol, tetrahydrofuran (THF), and water. THF and methanol are used as valuable organic solvents in chemical, pharmaceutical, and biochemical industries^6,7^. Also, THF has been proposed as a sustainable biofuel for internal combustion engines^8^. Achieving the required purity for these solvents is very important to the consumer^9^. The ternary mixture of MeOH/THF/water is produced in some industries such as polyvinyl chloride plants and magnetic film factories^10^. The mixture has two binary azeotropes: (1) THF/water and (2) MeOH/ THF. This makes it challenging to purify these components in ordinary distillation columns. Extractive distillation (ED) processes are usually used for the separation of azeotropic systems^11,12^. For instance, Shi et al. studied the separation of ternary azeotropic mixtures of isopropyl alcohol/isopropyl acetate/water through two alternative ED processes^13^. In ED methods, a solvent is employed to dissolve azeotropic components, so that the distillation curves are displaced in a way that the separation is facilitated. Ethylene glycol, diethylene glycol, and dimethyl sulfoxide (DMSO) are usually used as ED solvents^14,15^. Guo et al. investigated and optimized the ED process for the separation of the THF/methanol/water system^16^. They chose DMSO solvent and applied thermal integration, intermediate reboiler, and vapor re-compression methods to reduce the process operational cost. They showed that all their proposed methods for energy saving reduced the energy consumption of the ED process. For instance, the double-effect heat integration decreased the total annual cost (TAC) by about 25%. Also, the ED equipped with vapor recompression had 30% less carbon dioxide emissions than that of the main ED process. Yang et al. designed and optimized an ED process for the separation of THF and ethyl acetate from an industrial wastewater stream. Their proposed system consisted of three distillation columns^17^. They showed that the indirect ED process with heat integration had the highest economic performance.

For azeotropic mixtures, separation systems are designed based on their azeotropic characteristics. Separation approaches include pressure swing distillation, azeotropic distillation, extractive distillation, reactive distillation, or a combination of these methods^18–21^. Reactive distillation systems have been used by researchers in a variety of ways. In some cases, reactive distillation systems have been used alone, without any other methods^22–25^. In other cases, reactive distillation systems have been used in combination with other methods, such as reactive-extractive distillation (RED) and reactive distillation-pressure swing^26–29^. Su et al. studied the separation of the water/THF/ethanol mixture by reactive/extractive distillation process^30^. They proposed a systematic scheme for designing and optimizing RED to separate the ternary azeotropic compounds. In their proposed process, the mixture is fed to a reactive distillation column in which it reacts with ethylene oxide, and water is converted completely to ethylene glycol. The pure stream of ethylene glycol leaves the column as the bottom product, and the top product which is a binary azeotropic mixture of ethanol and THF is separated by the extractive distillation with dimethyl sulfoxide (DMSO) as the solvent. They showed that the developed process significantly reduces the total annual cost and carbon dioxide emissions compared to the pressure swing distillation process. Yang et al. also worked on the separation of the same azeotropic system by using the reactive distillation/pressure swing method^31^. In addition, they performed thermal integration to reduce energy consumption. The results of their research showed that the total annual cost and carbon dioxide emissions of the proposed process were reduced respectively by 51.8% and 59.1% compared to those of three-column pressure swing distillation. Kaymak studied the purification of bioethanol from fermentation broth based on the concept of the reaction of water with ethylene oxide to produce ethylene glycol. In this way, pure bioethanol can be produced without using any entrainer^32^. The results indicated that the designed process was economically superior to the conventional ED process, and the production of ethylene glycol generated additional revenue. Zhang et al. conducted a study on the separation of ternary mixtures containing water using reactive-extractive distillation on the following systems: 1-tert-butyl alcohol/water/ethanol, 2-tetrahydrofuran/water/ethanol, and 3-acetonitrile/isopropyl alcohol/water^33^. They concluded that the RED designs with two-column and three-column configurations have lower annual and operating costs compared to the extractive distillation method. Zhao et al. examined the separation process of the azeotropic mixture of isopropyl ether/isopropanol/water^34^. They designed three separation configurations: (1) reactive-extractive distillation, (2) reactive divided- wall column, and (3) reactive pressure-swing distillation. Their simulation results proved that the reactive- pressure-swing distillation was the best configuration.

So far, the undertaken efforts for the separation of water/methanol/tetrahydrofuran have involved extractive and pressure swing distillation methods. Reactive/extractive distillation methods have not yet been used in previous works to separate the water/methanol/tetrahydrofuran azeotropic system. This paper presents a novel approach of reactive/extractive distillation for separation of this mixture. In the following sections, we will propose two unique RED configurations for this mixture in this study. The newly proposed configurations consist of three distillation columns. The first column is a reactive distillation column in which ethylene oxide reacts with water to produce ethylene glycol. The bottom product of this column is pure ethylene glycol, and the top product is a binary azeotropic mixture of methanol and THF. The second and third columns are extractive distillation columns that are used to separate the methanol-THF mixture. Dimethyl sulfoxide and ethylene glycol are the two solvents that are investigated independently in the extractive distillation section. The economic and environmental indices of the designed RED configurations were calculated and compared with those of the conventional ED method.

## RED design methodology

In this research, the conventional method for separating the water/methanol/tetrahydrofuran azeotropic system is shortly called the “*base de*sign”, and our newly proposed configurations are named the “new designs”. The base design in this research is founded on the studies conducted by Gu et al.^16^. They used an extractive distillation method with dimethyl sulfoxide (DMSO) as the extracting solvent to separate components from three distillation columns^16^. The feed composition was defined based on the effluent stream of a chemical plant located in China. The feed temperature is 64.5 °C, and it has 25% THF, 37.5% Methanol, 37.5% water and a total molar rate of 500 kmol/h. The base design in this research is solely presented for the purpose of comparing its economic and environmental aspects with the proposed new designs.

**Figure 1:**
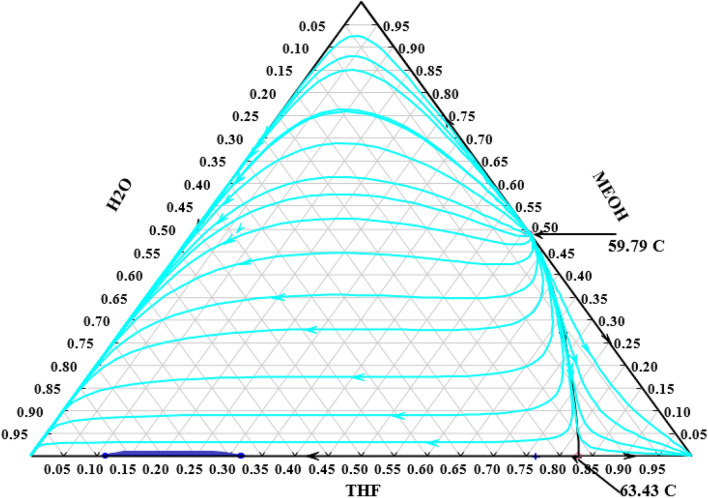
Distillation curves for water/methanol/THF mixture at atmospheric pressure.

Figure 1 illustrates the distillation curves of the water/methanol/THF system. Since there are two binary azeotropes it is not possible to separate this system by ordinary distillation columns.

In the following, the new designs are described, and its operating and equipment specifications are determined. Also, the design methodology and economic/environmental criteria are explained. Finally, the new designs are compared with the base design developed by Gu et. al.^16^.

### RED design algorithm

In the suggested RED designs, the first column is a reactive distillation (RD) column in which ethylene oxide reacts with water to produce ethylene glycol. In this column, water is completely converted to ethylene glycol which is removed as the bottom product. The top product is a binary azeotropic mixture of methanol/THF. Equations (1) and (2) present the stoichiometry and kinetics of the ethylene oxide hydrolysis reaction. The rate equation given in Eq. (2) were used and verified by some researchers^30,35^.

1 $${\text{EO}}+{\text{Water}}\to {\text{EG}}$$

2 $${\text{r}}\left(\frac{{\text{kmol}}}{{{\text{m}}}^{3}{\text{s}}}\right)=3.15\times {10}^{12}{\text{exp}}\left(-\frac{9547}{{\text{T}}}\right){{\text{X}}}_{{\text{EO}}}{{\text{X}}}_{{\text{Water}}}$$

**Figure 2:**
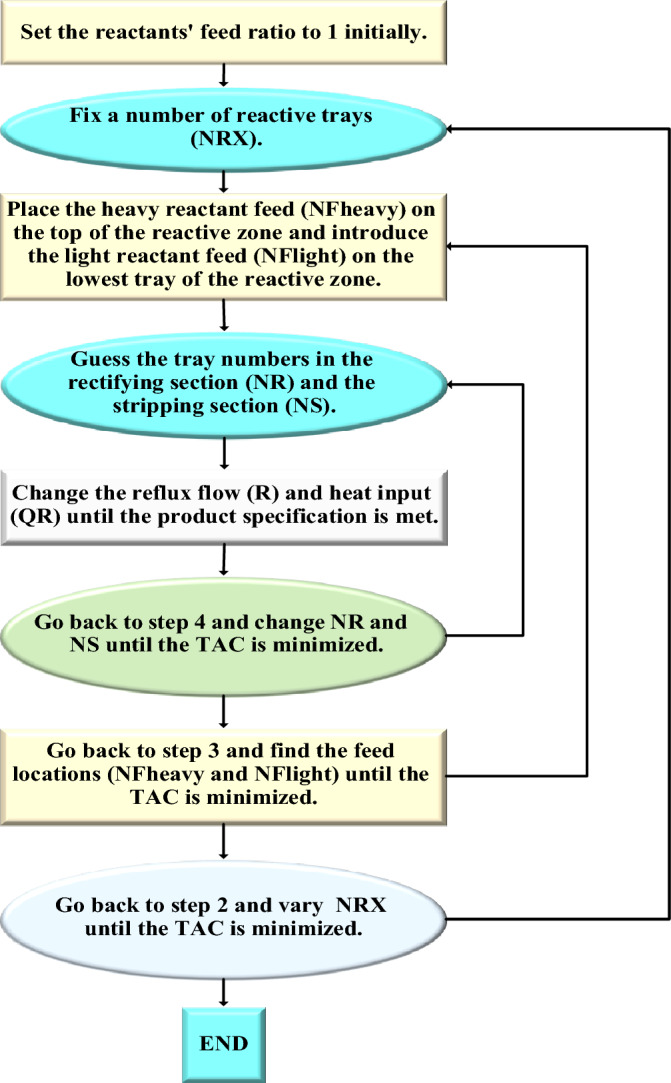
Luyben’s method for designing RD columns.

Luyben proposed an algorithm for RD columns, which is shown in Fig. 2^36^. We used this algorithm to design the first column of our proposed RED processes. According to this method, in the first stage, the ratio of ethylene oxide to water in the feed is set to match the stoichiometric ratio. For conceptual design purposes this assumption makes sense, but during the detailed process design, the actual ethylene oxide flow rate is usually considered slightly less than the stoichiometric amount to ensure its complete consumption in the reaction.

In the second stage, the number of reaction trays is estimated, and ethylene oxide and the main feed are introduced into the column at the bottom and top of the reaction zone, respectively. Then, the number of trays in the rectification and stripping sections is determined through a trial-and-error approach to minimize the total annual cost. In each iteration, the reflux ratio of the column is adjusted to achieve the required purity in the ethylene glycol product. In the final stage, the number of trays in the reaction section is modified, and the iterations are repeated to ultimately achieve the optimal design.

It should be noted that at each stage of Luyben’s algorithm the liquid holdups on the reaction zone trays are required. A trial-and-error method was used to calculate the liquid holdup on the reactive distillation column. First, the amount of holdup on the reaction trays is guessed. Then, the column is designed using Aspen Plus software and its diameter is obtained. Then, the holdup is calculated based on the column diameter and the default value of the weir height. The trial-and-error procedure continues until the difference between the guessed and the calculated values is less than a specified limit.

**Figure 3:**
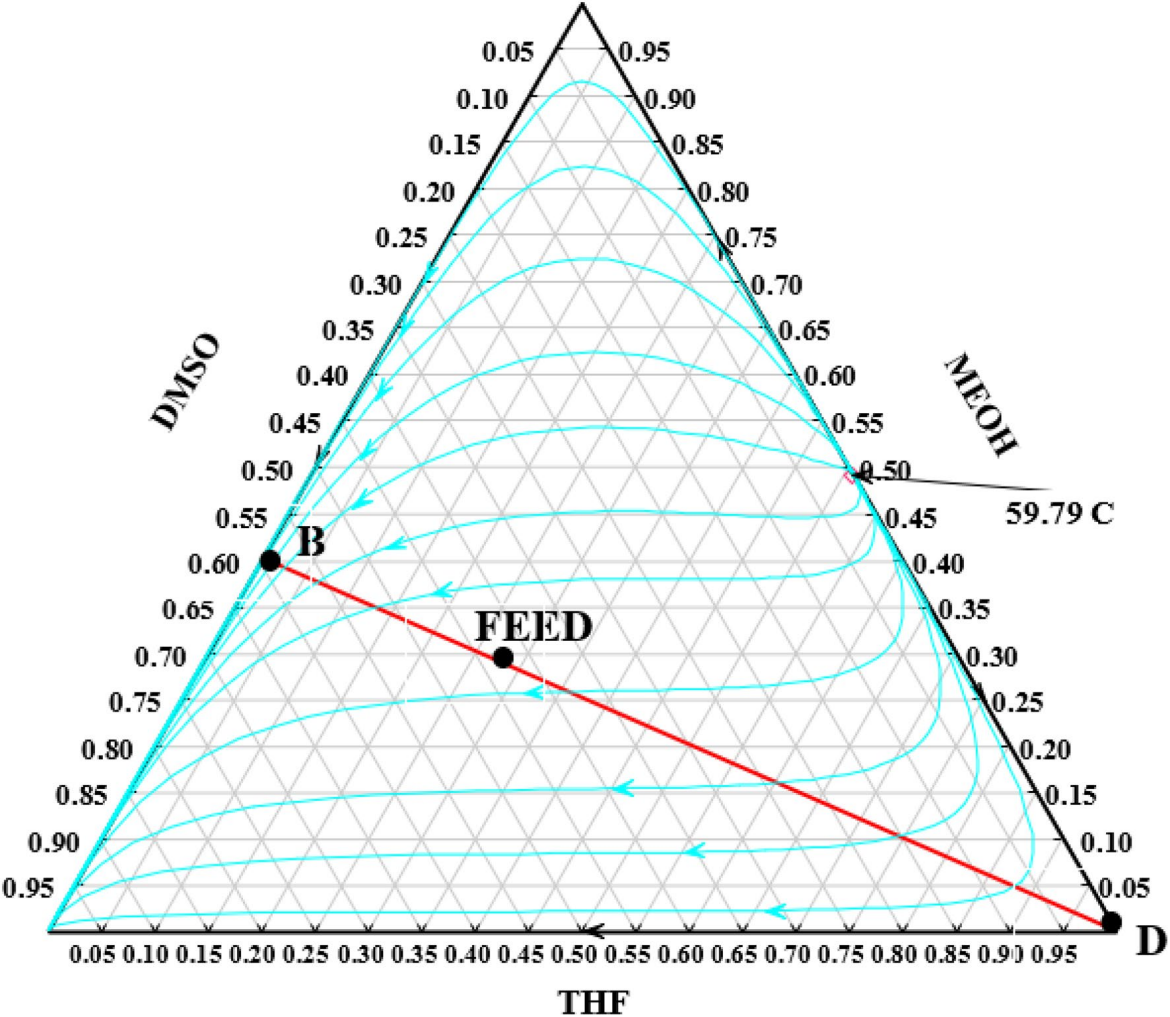
The residue curves of water/methanol/DMSO mixture at atmospheric pressure.

**Figure 4:**
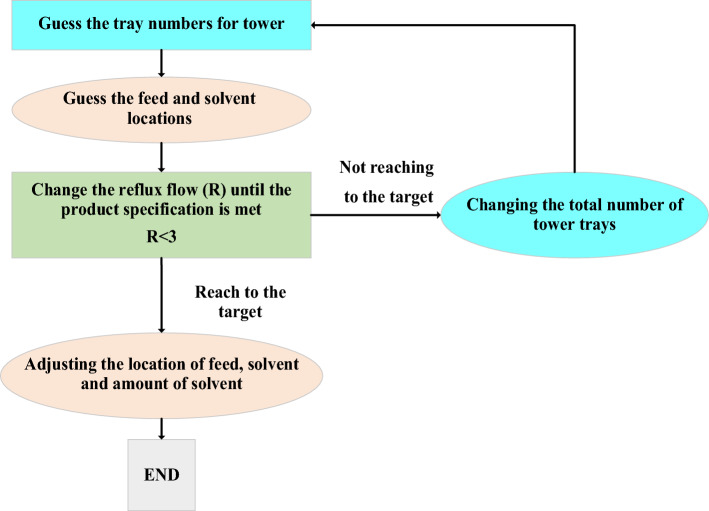
The approach used for the design of the extractive column.

In this work two RED designs were studied which differs in the solvent used for extractive distillation section. In the first new design, the top product of the RD column is separated by the ED method with DMSO as the solvent. The ED consists of two distillation columns: the extractive distillation column and the ordinary distillation (OD) column. In the extractive column pure THF is produced as the top product. The bottom product, which is a mixture of DMSO and methanol, does not have an azeotrope, and can be separated in an ordinary distillation column. The shortcut design of the extractive column can be performed by ConSep in Aspen Plus, but this method did not lead to a feasible design. The problem with this approach is that it is based on the residue curves shown in Fig. 3 which does not lead to the pure THF product as a feasible composition. Hence, the procedure illustrated in Fig. 4 was implemented to design this column. The column was simulated in Aspen plus with Radfrac which is based on a rigorous model of distillation columns.

**Figure 5:**
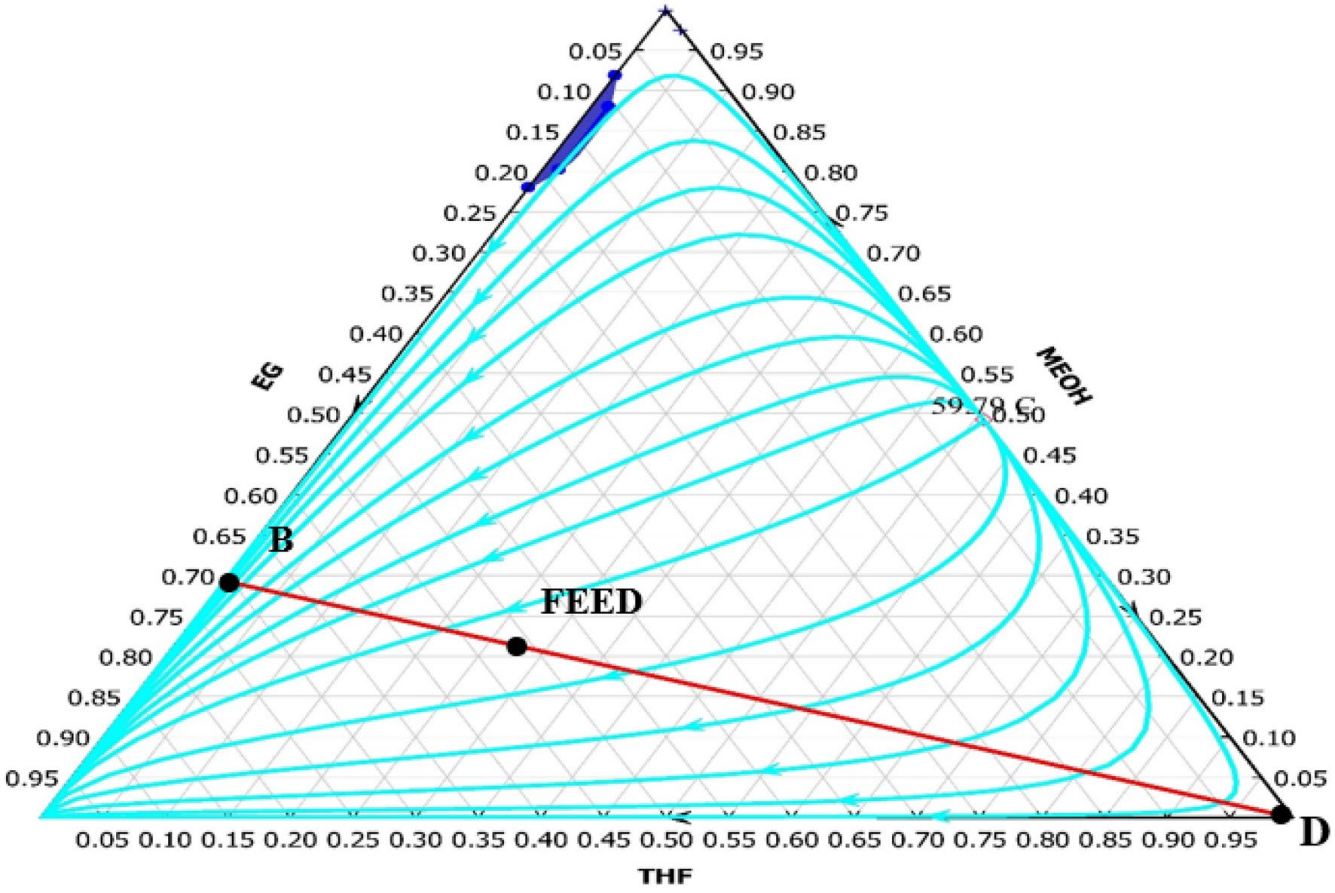
The residue curves of THF/methanol/EG mixture at atmospheric pressure.

The new design (II), like the new design (I), has three columns: RD, ED, and OD. However, ethylene glycol is employed instead of DMSO solvent in the new design (II). The ED column of this design cannot be designed with shortcut method too. This is also due to the form of the residue curves for the mixture of THF/methanol/EG as shown in Fig. 5. Hence, this column was designed by the algorithm shown in Fig. 4 using Radfrac rigorous model in Aspen Plus.

In both new designs, the ordinary columns for the separation of the methanol/DMSO or methanol/EG mixtures were designed according to the usual method. In this way, an initial number of trays, feed tray, and the reflux ratio are determined by a FUG shortcut model, and then these parameters are fine-tuned by applying the rigorous model of Radfrac.

### Economic criteria

In the design of industrial units, engineers generally explore a design with the lowest operating and investment costs. The objective function used during the optimization of the proposed designs is the total annual cost (TAC). TAC incorporates both operating and investment costs of the process. Therefore, in this study, the total operating cost, the total capital cost, and finally the total annual cost, were calculated and compared separately to show the economic advantages of the suggested new designs. The calculation of the total capital cost (TCC) is based on Eqs. (3–7)^37^.

As per Eq. (3), the total capital cost (TCC) is the summation of the heat exchanger and the tower costs.

3 $${\text{TCC}}={{\text{HX}}}_{{\text{Cost}}}+{\mathrm{ Towers}}_{{\text{Cost}}}$$

The heat exchanger cost, HX_Cost_ includes the cost of reboilers, condensers, and coolers, and it is estimated based on the area of total heat transfer area as given in Eq. (4).

4 $${{\text{HX}}}_{{\text{Cost}}}=(\mathrm{Reboilers } \; \& \; {{\text{Condensers}} \; \& \; {\text{Cooler}})}_{{\text{Cost}}}=7296{\left({\text{area}}\right)}^{0.65}$$

In the above equation, the area is in square meters and is calculated as below:

5 $${\text{A}}=\frac{{\text{Q}}}{{\text{U}}\times \Delta {\text{T}}}$$

where Q is the exchanger duty in kW, U is the overall heat transfer coefficient in kW/(K.m^2^) and ∆T is the mean temperature difference in K. Typical values for U in reboilers, condensers, and coolers are respectively, 0.568 kW/(K.m^2^), 0.853 kW/(K.m^2^), and 0.5 kW/(K.m^2^). The average temperature difference between hot and cold streams, ∆T, is calculated based on the log mean temperature difference (LMTD).

The tower cost can be calculated according to Eq. (6).

6 $${{\text{Towers}}}_{{\text{Cost}}}=17640 {\left({\text{D}}\right)}^{1.066}{\left({\text{L}}\right)}^{0.802}$$

where D and L are respectively the tower diameter and height in meters. If N_T_ is the number of stages, then the tower height, L, is estimated as the following:

7 $${\text{L}}=1.2\times 0.61\times \left({{\text{N}}}_{{\text{T}}}-2\right)$$

Equations (8–10) are used for estimating the total operating cost (TOC). The TOC consists of the cost of the steam used in the reboilers (SC), the cost of make-up solvent (MSC) in the extractive distillation column, and the cost of ethylene oxide (EOC) in the reactive distillation column.

8 $$ {\text{TOC }} = {\text{ SC }} + {\text{ MSC }} + {\text{ EOC}} $$

According to Eq. (9), the steam cost (SC) depends on the reboiler duty (Q) and the steam type (LP, MP, and HP steam).

9 $${\text{SC}}={\text{Q}}\times \mathfrak{J}\times \frac{3600\;{\text{S}}}{{\text{h}}}\times \frac{24\;{\text{h}}}{{\text{day}}}\times \frac{335\;{\text{day}}}{{\text{yer}}}$$

The unit of $$\mathfrak{J}$$ is $/GJ and its value is related to the type of steam^37^. The cost of the raw materials (solvents and ethylene oxide) can be computed by the following equation.

10 $$\mathrm{MSC\,or\,EOC}={\text{m}}\times \epsilon \times \frac{24\;{\text{h}}}{{\text{day}}}\times \frac{335\;{\text{day}}}{{\text{yer}}}$$

“m” is the mass flow rate (kg/s) and “ε” represents the price of the material in $/kg. The prices of DMSO, EG, and ethylene oxide are respectively 4.64 $/kg, 0.85$/kg, and 1 $/kg^24^.

Considering a three-year payback period, TAC is described by Eq. (11).

11 $${\text{TAC}}=\frac{{\text{TCC}}}{\mathrm{Payback \; Period}}+{\text{TOC}}$$

### Environmental indices

The new designs suggested in this research is intended for the reduction of carbon dioxide emissions. Equation (12) is applied to calculate the amount of carbon dioxide emissions^38^.

12 $${{\text{CO}}}_{2}\;\mathrm{ emissions}=\left(\frac{{{\text{Q}}}_{{\text{fuel}}}}{{\text{NHV}}}\right)\times \left(\frac{\mathrm{C\%}}{100}\right)\mathrm{\alpha }$$

In Eq. (12), Q_fuel_ is the amount of energy consumed in the reboilers in kJ/h, and NHV is the net heating value of the fuel (39,771 kJ/kg). The carbon percentage in the fuel, %C, has been considered 86.5. Also, “α” is the ratio of CO_2_ molecular weight to carbon atomic weight, which is 3.67.

### Modeling and simulation

In this research, it is assumed that the process is at steady state conditions, and the heat loss and the pressure drop in the equipment items are negligible. Also, according to the characteristics of the components in the process, the NRTL activity model has been used for VLE calculations.

The validity of the NRTL model were explored in some research works^39,40^, and its binary interaction coefficients are presented in Table A at the appendix. In this section, the process flow diagrams of the base design and the proposed new designs are illustrated and explained.

#### The base design

**Figure 6:**
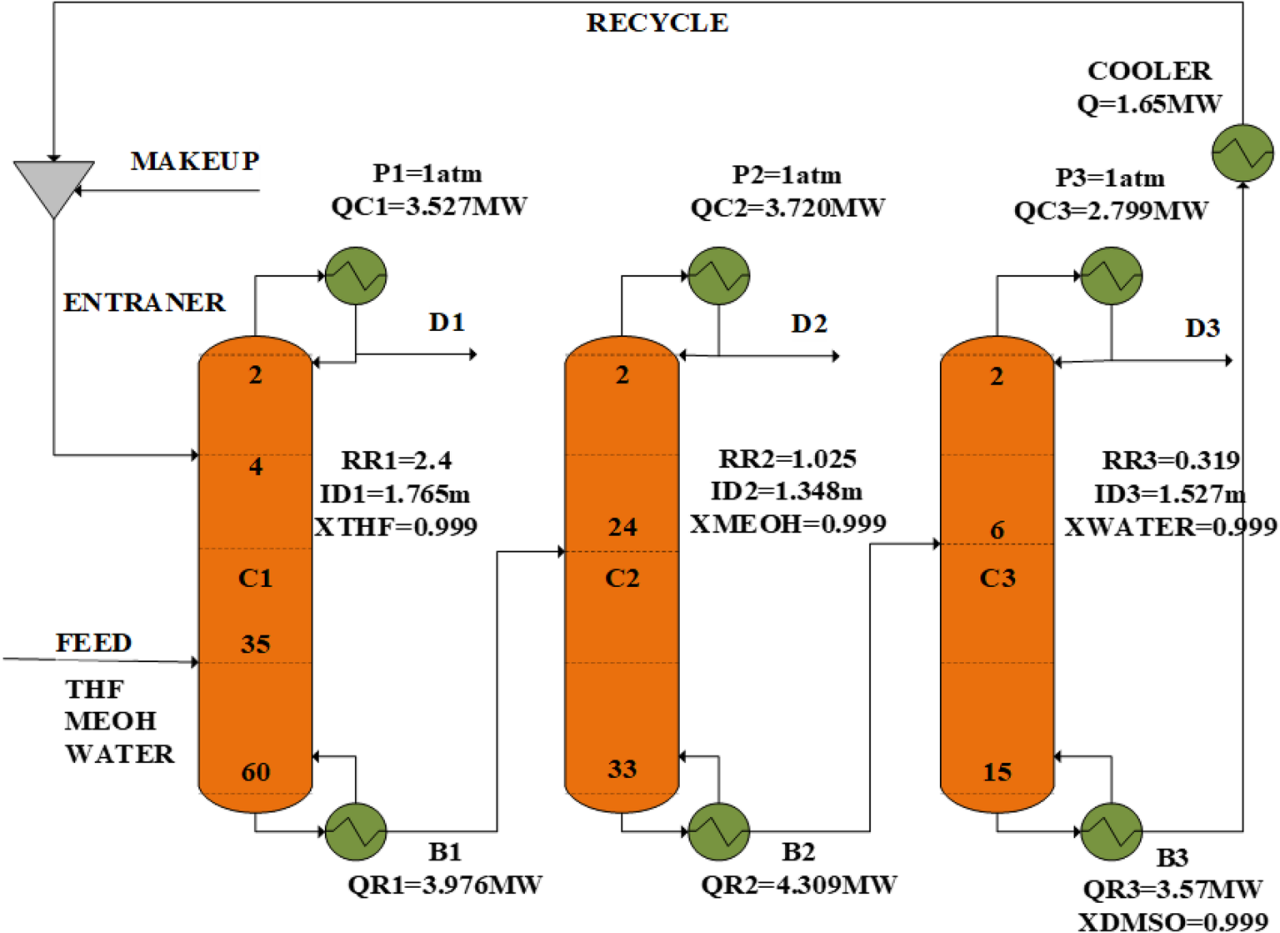
The process flow diagram of the base design.

The extractive distillation method has been used to separate the ternary azeotropic system of water/methanol/tetrahydrofuran. Figure 6 shows the process flow diagram of this process as proposed by Gu et al.^16^.

They used highly pure DMSO as the solvent, and there are three columns. The feed stream is entered into the first column on tray 35 at 1 atm pressure and 64.5 °C temperature. The solvent DMSO goes into this column on tray 4, and the pure THF stream (99.9%) is separated as the top product. The bottom product, which contains methanol, water, and DMSO, is fed into the 2nd column on tray 24. This column separates the pure methanol stream (99.9%) as the top product. The bottom product of the second column is sent to the third distillation column for purification of DMSO. The DMSO stream from the bottom of the 3rd column is recycled to the first column. The recycled DMSO is cooled to 60 °C before entering the 4th tray of the first column. A small amount of DMSO is lost during the process, thus to make up for it, another DMSO stream has been added to the recycling flow.

**Figure 7:**
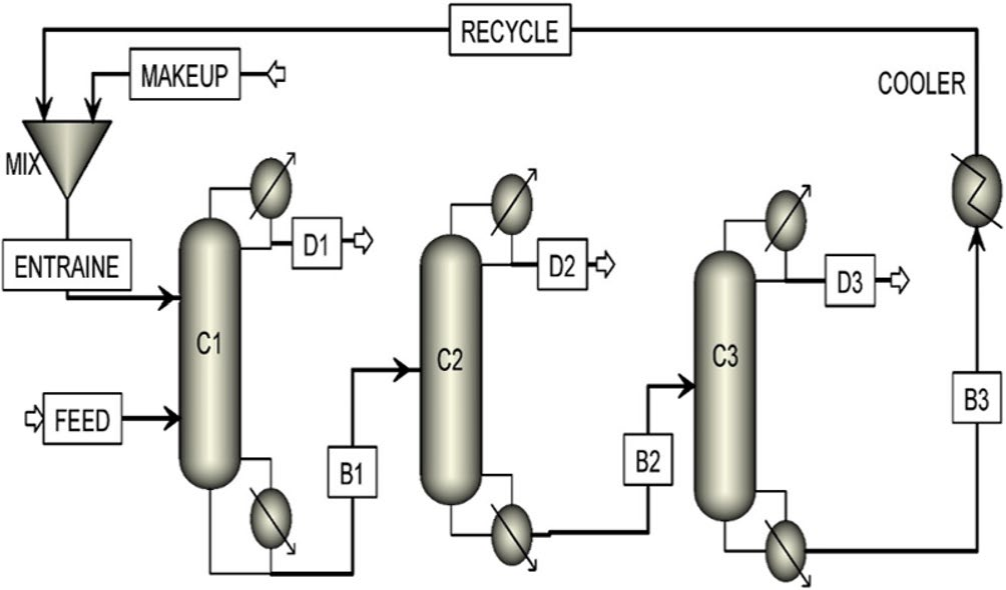
Simulation flow diagram of the base design.

The simulation flow diagram is illustrated in Fig. 7, and the operating conditions obtained from the simulation by AspenPlus are given in Table 1.

**Table 1:** The operating conditions of the streams in the base design.

Stream	Vapor fraction	Temperature (°C)	Mole flow (kmol/h)	Composition (mole%)			
				THF	MEOH	H_2_O	DMSO
Feed	0	64.5	500.0	25.000	37.500	37.500	0.000
Entrainer	0	60.0	290.5	0.000	0.000	0.010	99.990
D1	0	65.9	125.1	99.919	00.076	Negligible	Negligible
B1	0	96.1	665.4	Negligible	28.164	28.180	43.655
D2	0	64.5	187.5	Negligible	99.940	00.060	Negligible
B2	0	137.7	477.9	Negligible	Negligible	39.212	60.783
D3	0	100.0	187.5	0.000	00.010	99.930	00.060
B3	0	190.7	290.4	0.000	Negligible	Negligible	99.995
Recycle	0	60.0	290.4	0.000	Negligible	Negligible	99.995
Makeup	0	190.7	0.1	0.000	0.000	0.000	1.000

#### The new design (I)

**Figure 8:**
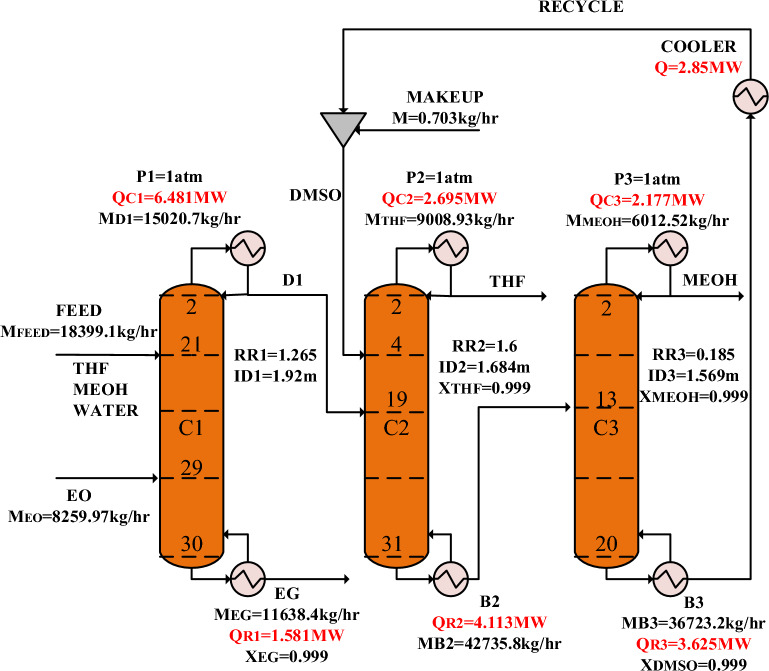
The process flow diagram of the new design (I).

The process flow diagram of the new design (I) proposed in this research, which is based on the reactive/extractive distillation method (RED) with DMSO solvent is shown in Fig. 8. This process has three distillation columns: (1) the reactive distillation (RD) column, (2) the extractive distillation (ED) column, and (3) the ordinary distillation (OD) column.

The main feed and ethylene oxide streams are entered into the RD column. Ethylene oxide is a volatile component with a normal boiling point of 10 °C, so it moves to the top of the column in the form of vapor. Ethylene oxide reacts with water and produces the valuable by-product of ethylene glycol. In this way, water is removed from the ternary azeotropic mixture. The pure ethylene glycol is separated as the bottom product. The top product, which is a mixture of THF and methanol, is fed into the ED column. The ED column’s feed has a minimum boiling azeotrope at 59.8 °C and 1 atm. Hence, DMSO is used as the solvent to separate methanol from THF. The solvent stream is entered into the top of the ED column, and the pure THF (99.9%) is removed as the distillate product. The bottom product of the ED column is sent to the last column for recovering the solvent DMSO. In this column, pure methanol (99.9%) is separated as the top product. The bottom product, which is the recovered DMSO solvent, is recycled to the ED column after passing through a cooler. To compensate for the loss of the DMSO solvent in the process, a make-up stream is added to the recycle stream.

#### The new design (II)

**Figure 9:**
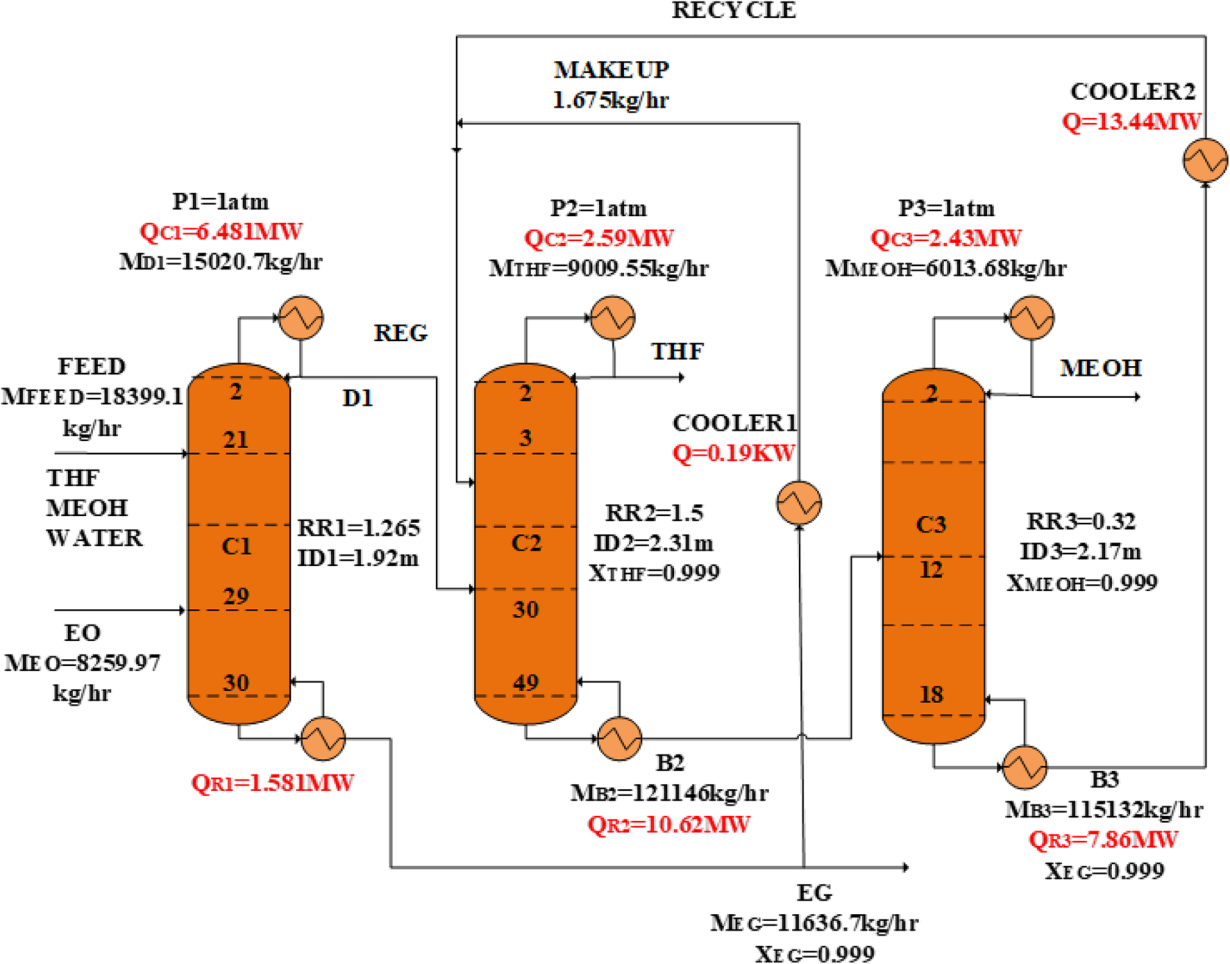
The process flow diagram of the new design (II).

The new design (II) with the process flow diagram in Fig. 9 is similar to the new design (I), and consists of RD, ED, and OD columns. In the RD column, which is designed like the first column of the design (I), a mixture of THF and methanol is obtained from the top of the column, and the ethylene glycol with a purity of 99.9% is removed from the bottom of the column. The THF/methanol mixture is fed to the extractive distillation column, and THF is purified with the help of EG solvent. Methanol is dissolved in EG and goes to the bottom product. The methanol/EG mixture is separated in the ordinary distillation column from the top and bottom, respectively, with a purity of 99.9%. The EG solvent from the bottom of the OD column, after cooling in the cooler, returns to the ED column. In this design, part of the ethylene glycol product obtained in the first column is used as a make-up stream to compensate for the lost solvent.

#### Design procedure

The design procedure consists of three parts: (1) designing the RD column based on Luyben’s method as described in Fig. 2, (2) applying the method shown in Fig. 4 to the ED column, and (3) using FUG shortcut technique for the OD column. The results of the simulation and optimization of the proposed new designs are presented and discussed in the subsequent sections.

## Results and discussion

### Simulation and optimization of the new design (I)

This section discusses the results of the design and optimization of the columns of the new design (I). These columns include the reactive distillation column (RDC), the extractive distillation (ED) and ordinary distillation (OD) columns.

#### The RDC design and optimization

In Luyben's algorithm, the first step involves estimating the number of trays in the reaction section of the column. Initially, nine trays were considered for the column reaction section. This is followed by estimating and optimizing the number of trays in the rectification and stripping sections. The third step involves shifting the reaction area to specify the best situation for the feed trays. The number of reaction trays is then improved and reevaluated. The results of each of these stages are explained in detail below.

**Figure 10:**
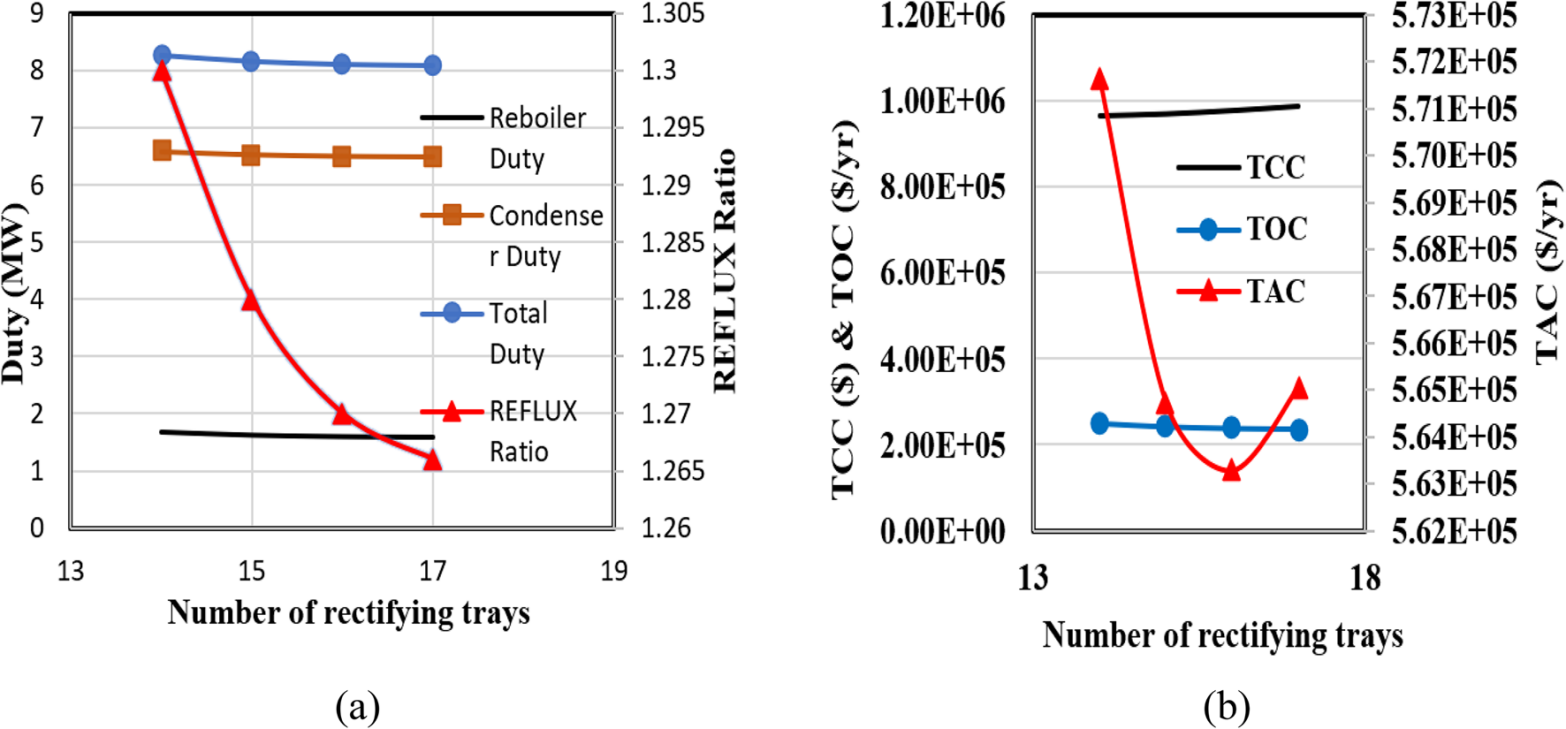
The calculations for the rectifying section of the RD column in the new design (I).

Figure 10a depicts the relationship between the number of rectifying trays and the reflux ratio, the reboiler and condenser duties for the RD column. The diagrams demonstrate a consistent decrease in all operating parameters as the number of trays increases. Furthermore, Fig. 10b illustrates the variations in capital, operating, and total annual costs in terms of the number of trays in the rectifying section. The point of minimum TAC is observed at 16 trays.

**Figure 11:**
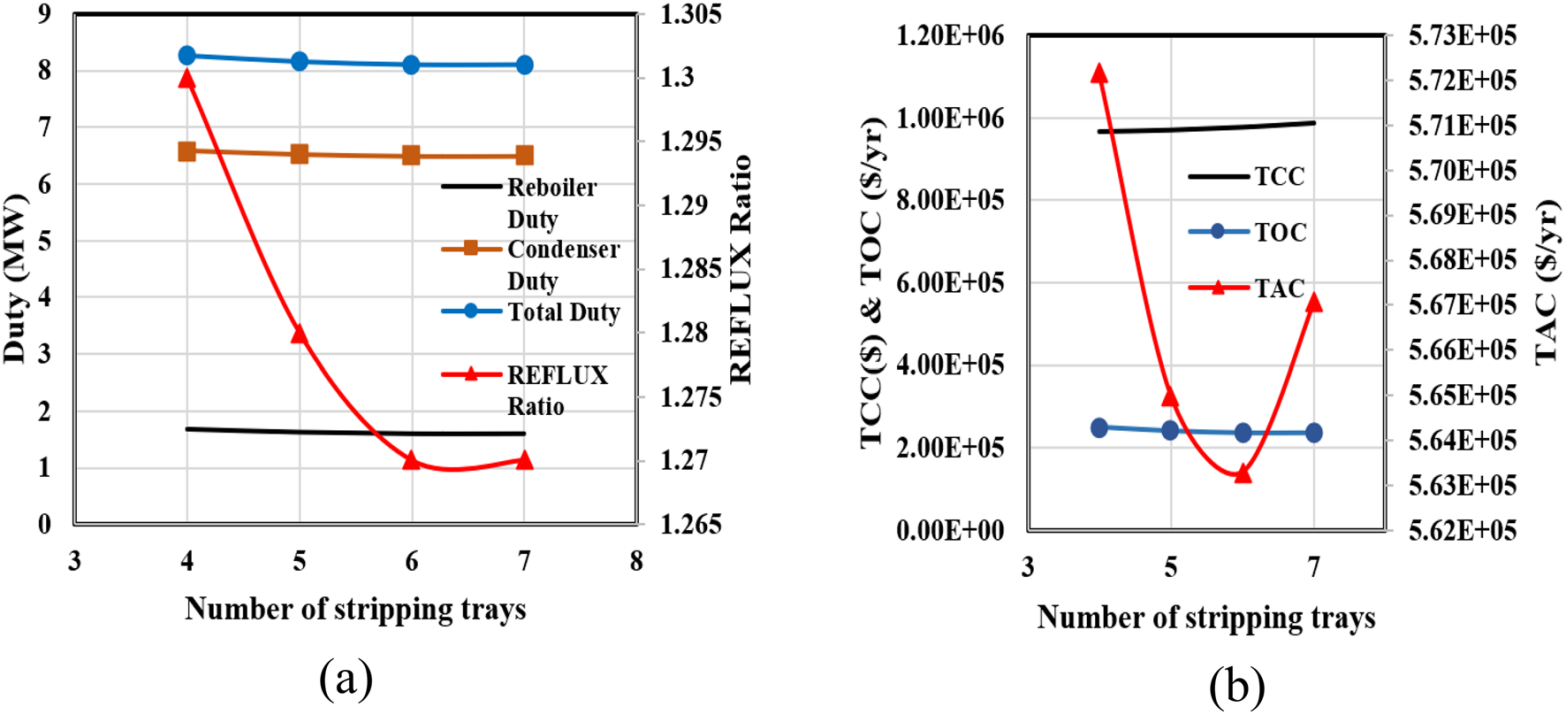
The results of calculations for the stripping section of the RD column for the new design (I).

Figure 11 illustrates that the reflux ratio diminishes as the number of trays in the stripping section increases. This decline persists until reaching six trays, marking a point where the reflux ratio stabilizes. This phenomenon leads to a reduction in the thermal loads of the reboiler and condenser, followed by a stabilization of these loads. Consequently, total energy consumption and overall operating costs follow a descending trajectory, eventually converging to a steady level at the seventh tray. Nonetheless, adding more trays to the distillation column comes with an increased capital cost, resulting in a point of minimum TAC when the stripper section has six trays.

**Figure 12:**
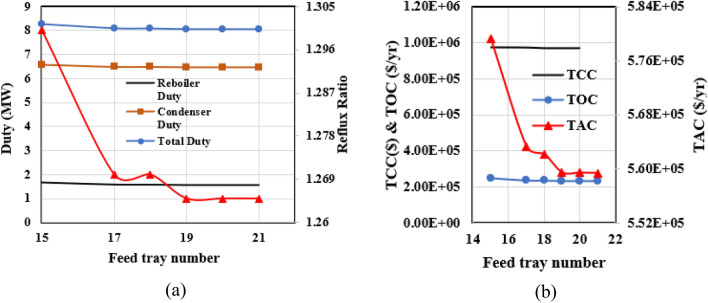
The calculation results for shifting the RD column's reaction section for the new design (I).

The third phase in Luyben's approach involves investigating the shifting of the reaction zone within the RD column. To accomplish this, the count of reaction trays remains constant, while adjustments are made solely to the points of entry for the primary feed and the EO stream. It becomes possible to identify the ethylene oxide feed tray by designating the primary feed tray since there is a fixed number of trays in the reaction zone. Consequently, the x-axis in Fig. 12 corresponds to the tray number for the main feed. As depicted in Fig. 12a, the displacement of the reaction zone toward the column's base leads to a reduction in the reflux ratio, ultimately stabilizing at tray 21. The changes in the reboiler and condenser heat duties are consistent with the changes in operating costs; they get smaller as the feed tray descends the column and remains unchanged for feed trays located below tray 21.The capital cost of the RD column remains unaffected for two key reasons: firstly, the shift of the reaction zone assumes a constant total tray count within the tower, and secondly, minimal changes occur in the tower's diameter due to the slight decrease in the reflux ratio. Figure 12b illustrates the variation in the total annual cost concerning the placement of the main feed tray.

Shifting the reaction zone to the column's bottom results in a gradual reduction of the TAC until it stabilizes. This graphical representation highlights that trays 21 and 29 are optimal for the main feed and ethylene oxide flow, respectively.

**Figure 13:**
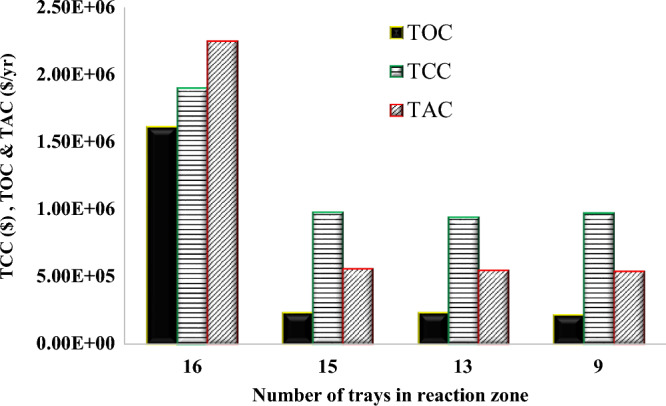
The effect of the number of reaction trays on economic indices of the new design (I).

To study the effect of the number of trays in the reaction zone on the economic parameters of the column, it was changed from 16 to 9. Figure 13 shows the effect of the number of reaction trays on the operating, capital, and total annual costs. The TAC is inversely proportional to the number of trays in the reaction section. The relationship is not linear but rather curves downward. It should be mentioned that in proportion to the increase in the number of reaction zone trays, the number of trays in the entire column and the rectification and stripping sections should be increased. Also, the feed tray numbers have been adjusted to cope with variations in the number of trays. The computation outcomes demonstrate a remarkable 75% reduction in the TAC by decreasing the count of reactive trays from 16 to 9. As evidenced by the diagram, the most economical TAC is achieved with nine trays. Based on the small differences (~ 1%) in TAC between 13 and 9 trays in the reaction zone, it can be concluded that selecting 9 trays is a suitable choice for the reaction zone of the RD column.

#### The design of the ED and OD columns

In the procedure of designing the ED column, it becomes necessary to make informed assumptions about the column's initial parameters. As a result, reference was made to the design parameters of a comparable ED column detailed in Su et al.'s research^30^. These parameters were then adjusted proportionally according to the column's feed flow rates, leading to the acquisition of appropriate initial estimations for the column parameters- the total tray count, solvent flow rate, feed and solvent tray locations. Subsequently, through an iterative calculation, the total tray count and the reflux ratio were established. This procedure led to the determination of 32 trays for the column and a reflux ratio of 1.6. Following this, the positions for introducing the main feed and solvent were fine-tuned. The 19th tray was identified as the most suitable for the main feed introduction, while the solvent was introduced at the 4th tray. In the last stage, the solvent flow rate entering the tower was determined to be 470 kmol/h.

The 3rd column which is used for the recovery of the solvent DMSO is an ordinary column and can be designed by the conventional procedure. At first, initial guesses were provided for the number of trays, the reflux ratio, and the feed tray by using the ASPEN shortcut FUG method. Then, the column was rigorously simulated by ASPEN’s Radfrac model. The initial reflux ratio was adjusted in the rigorous model to obtain pure methanol (99.9%) at the top product. The design results for the total number of trays, the feed tray, and the reflux ratio are respectively, 21, 13, and 0.18.

### Simulation and optimization of the new design (II)

In the new design (II), the RD column is completely similar to the new design (I), and the only difference is in the solvent used in the ED column. Therefore, in the design (II), the distillation columns, both extractive and ordinary, need to be redesigned. The method for doing this is like the technique used for these two columns in the design (I). The obtained parameters for these two columns are presented in Table 2.

**Table 2:** Design results of the extractive and ordinary columns of the design (II).

Equipment	Variable	Value
ED column (C_2_)	${{\text{N}}}_{{\text{T}}2}$	50
	${{\text{ID}}}_{2},{\text{m}}$	2.31
	${{\text{Q}}}_{{\text{C}}2},{\text{MW}}$	2.59
	${{\text{Q}}}_{{\text{R}}2},{\text{MW}}$	10.62
OD column (C_3_)	${{\text{N}}}_{{\text{T}}3}$	19
	${{\text{ID}}}_{3},{\text{m}}$	2.17
	${{\text{Q}}}_{{\text{C}}3},{\text{MW}}$	2.43
	${{\text{Q}}}_{{\text{R}}3},{\text{MW}}$	7.86
Cooler	${{\text{Q}}}_{{\text{Cooler}}},{\text{MW}}$	13.44
EG makeup	m, kg/h	1.67
EO flow	m, g/h	8259.97

### The simulation of the new design (I)

**Figure 14:**
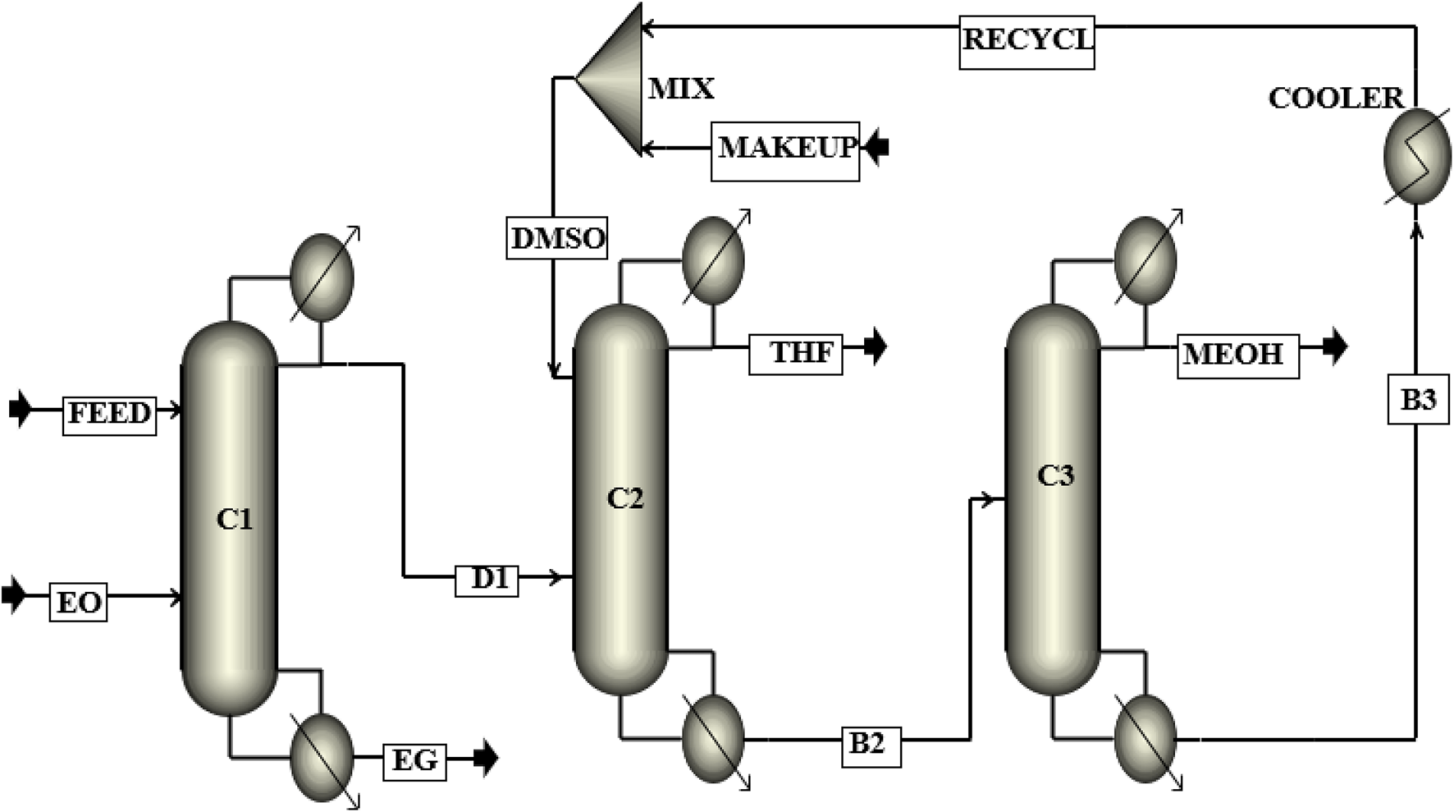
Simulation flow diagram of the new design (I).

The simulation flow diagram of the suggested RED process of the new design (I) is illustrated in Fig. 14, and the operating conditions of the streams for the optimum design of the process are presented in Table 3.

**Table 3:** Operating conditions of the main streams of the new design (I).

Stream	Vapor fraction	Temp. °C	Flow kmol/h	Composition (mole%)					
				THF	MEOH	H_2_O	DMSO	EO	EG
Feed	0	64.58	500.00	25.000	37.500	37.500	0.000	0.000	0.000
EO	1	10.34	187.50	0.000	0.000	0.000	0.000	100.000	0.000
D1	0	59.94	312.50	40.010	59.980	0.0100	0.000	0.000	0.000
EG	0	196.56	187.53	0.000	00.0200	0.000	0.000	00.019	99.960
DMSO	0	50.00	470.00	0.000	0.000	0.000	99.999	0.000	0.000
THF	0	65.93	125.00	99.900	0.089	0.000	0.000	0.000	0.000
B2	0	113.52	657.50	0.017	28.494	0.000	71.480	0.000	0.000
MEOH	0	64.52	187.50	0.060	99.910	0.019	0.000	0.000	0.000
B3	0	190.74	470.00	0.000	0.000	0.000	99.999	0.000	0.000
Recycle	0	50.00	470.00	0.000	0.000	0.000	99.999	0.000	0.000
Makeup	0	50.00	0.009	0.000	0.000	0.000	100.000	0.000	0.000

**Figure 15:**
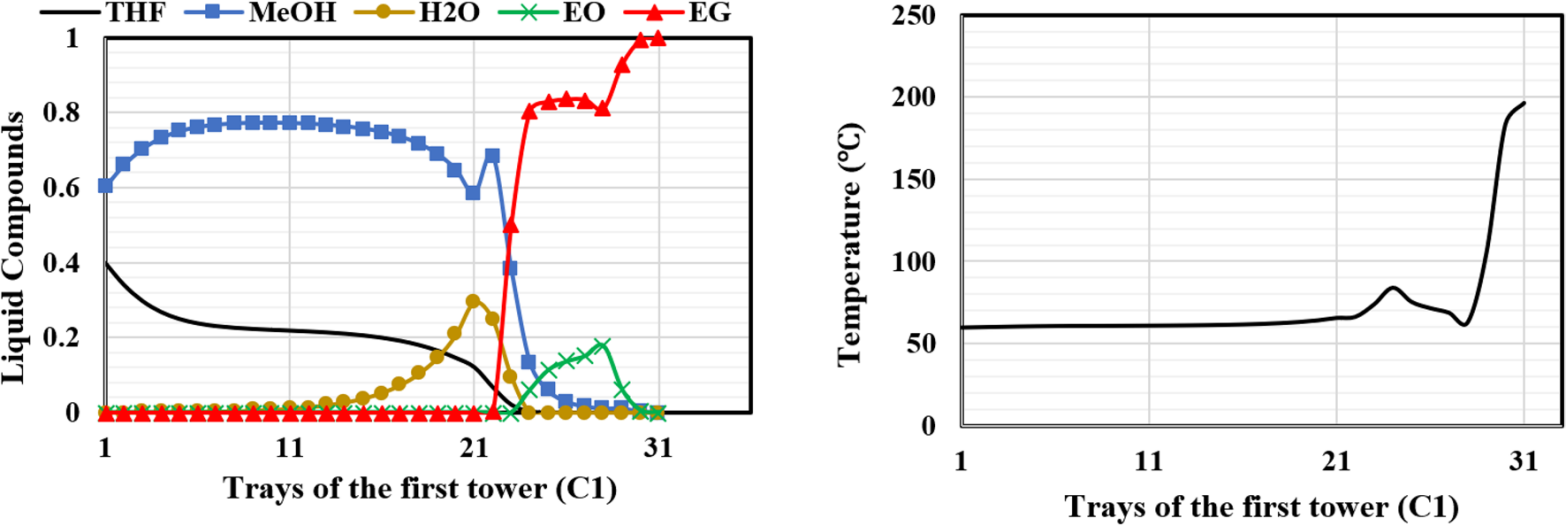
Temperature and composition profiles for RD (C1) column for design (I).

**Figure 16:**
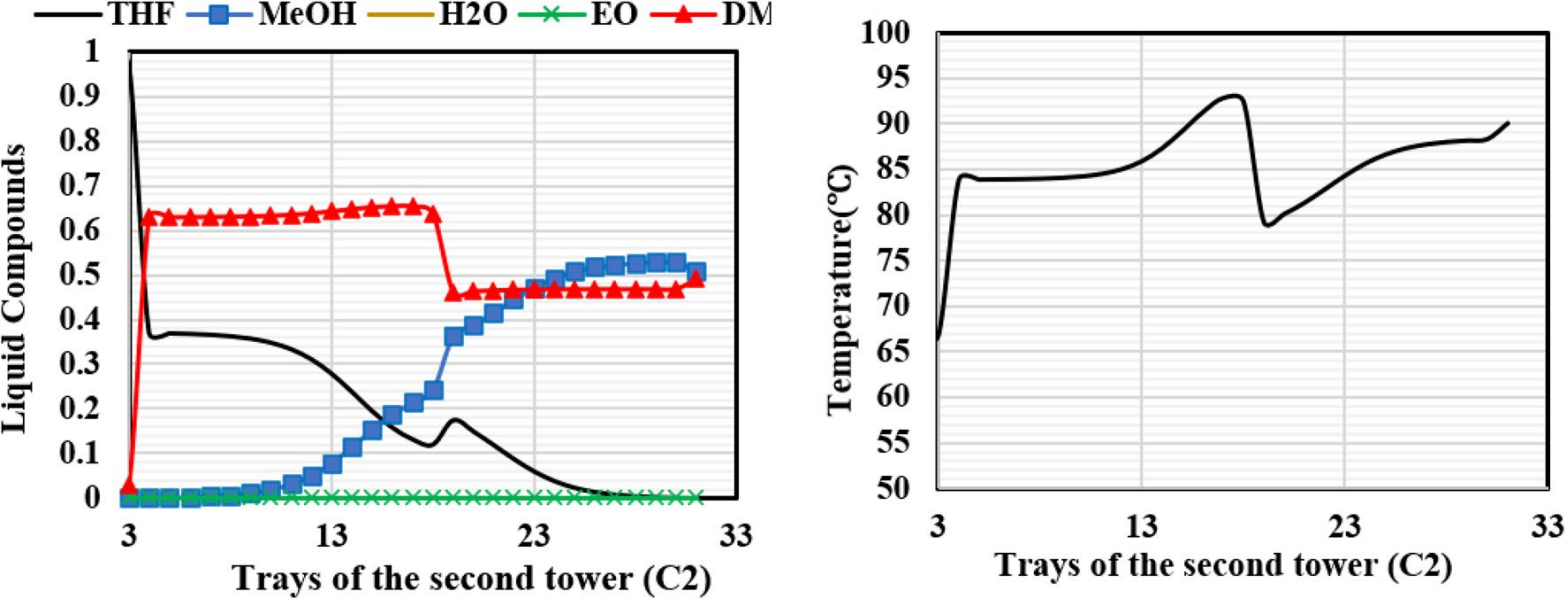
Temperature and composition profiles for ED (C2) column for design (I).

**Figure 17:**
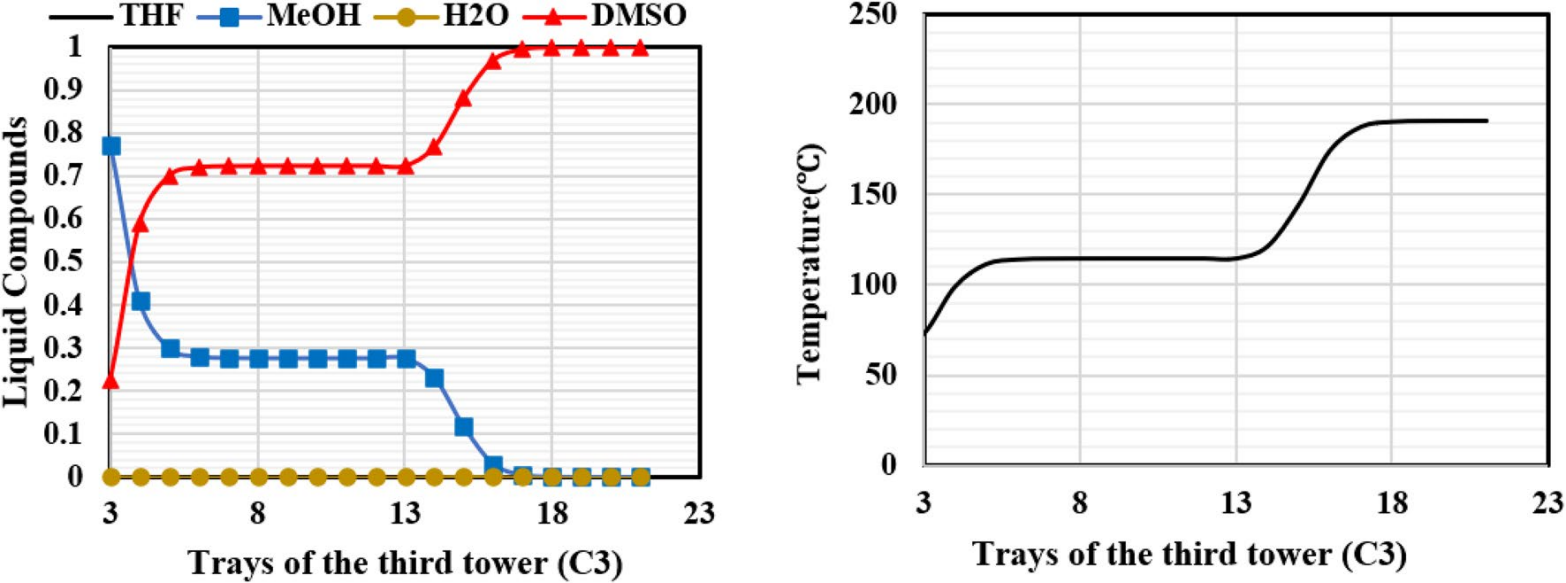
Temperature and composition profiles for OD (C3) column for design (I).

Figures 15, 16, 17 illustrate the composition and temperature profiles on the column trays in the liquid phase for each of the columns of the new design (I). As seen in Fig. 15, the ethylene oxide moves towards the top of the RD column, and its concentration shows an upward and downward trend. This is because ethylene oxide reacts with water to produce ethylene glycol. Ethylene oxide is completely consumed due to the reaction with water up to tray 23. This explanation also accounts for the decrease in the ethylene glycol concentration after tray 24. The pure ethylene glycol product moves to the bottom of the column because of its high boiling point. Also, the volatile mixture of methanol and tetrahydrofuran leaves the column as the distillate product. Due to the heat of the reaction, the temperature profile has a maximum in column C_1_ on tray 24.

As shown in Fig. 16, the temperature profile of column C2 shows a sharp rise and fall due to the introduction of the heavy solvent on tray 4 and the light mixture feed on tray 18. This is because the solvent and the mixture streams have different boiling points, and the introduction of these two streams causes a significant change in the composition of the liquid on the trays. The mixture of methanol and DMSO does not have an azeotrope, so they can be easily separated in the ordinary distillation column, C3 (Fig. 17).

### The simulation of the new design (II)

**Figure 18:**
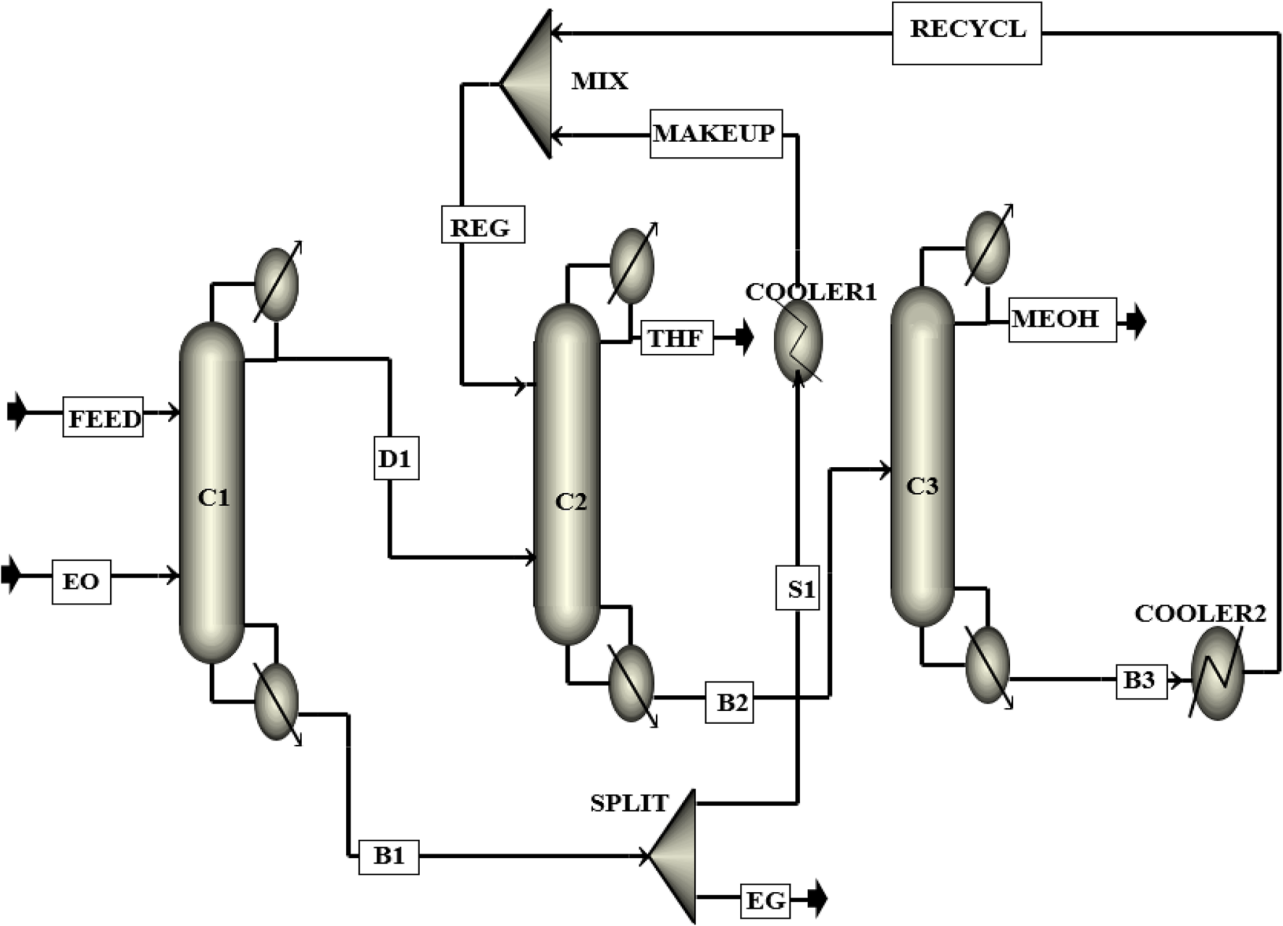
Simulation flow diagram of the new design (II).

The simulation flow diagram of the new design (II) is illustrated in Fig. 18, and its operating conditions are presented in Table 4.

**Table 4:** Operating conditions of the main streams of the new design (II).

Stream	Vapor fraction	Temp. (°C)	Mole flow (kmol/h)	Composition (mole%)				
				THF	MEOH	H_2_O	EO	EG
Feed	0.00	64.6	500.00	25.00	37.50	37.50	0.00	0.00
EO	1.00	10.3	187.50	0.00	0.00	0.00	100.00	0.00
D1	0.00	59.9	312.50	40.00	59.99	0.010	0.00	0.00
EG	0.00	196.6	187.51	0.00	0.019	0.00	0.019	99.96
B2	0.00	132.8	2037.52	0.00	0.20	0.00	0.00	90.80
THF	0.00	65.9	125.00	99.90	0.08	0.00	0.00	0.02
Makeup	0.00	50.0	0.03	0.00	0.02	0.00	0.0.02	99.96
MeOH	0.00	64.5	187.54	0.062	99.910	00.019	0.000	Negligible
Recycle	0.00	50.0	1850.00	0.00	0.00	0.00	0.000	100.00

**Figure 19:**
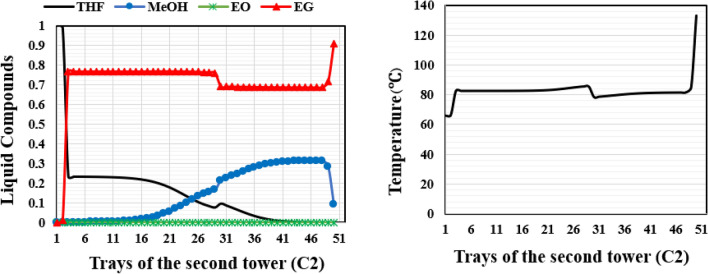
Temperature and composition profiles for ED (C2) column of the new design (II).

**Figure 20:**
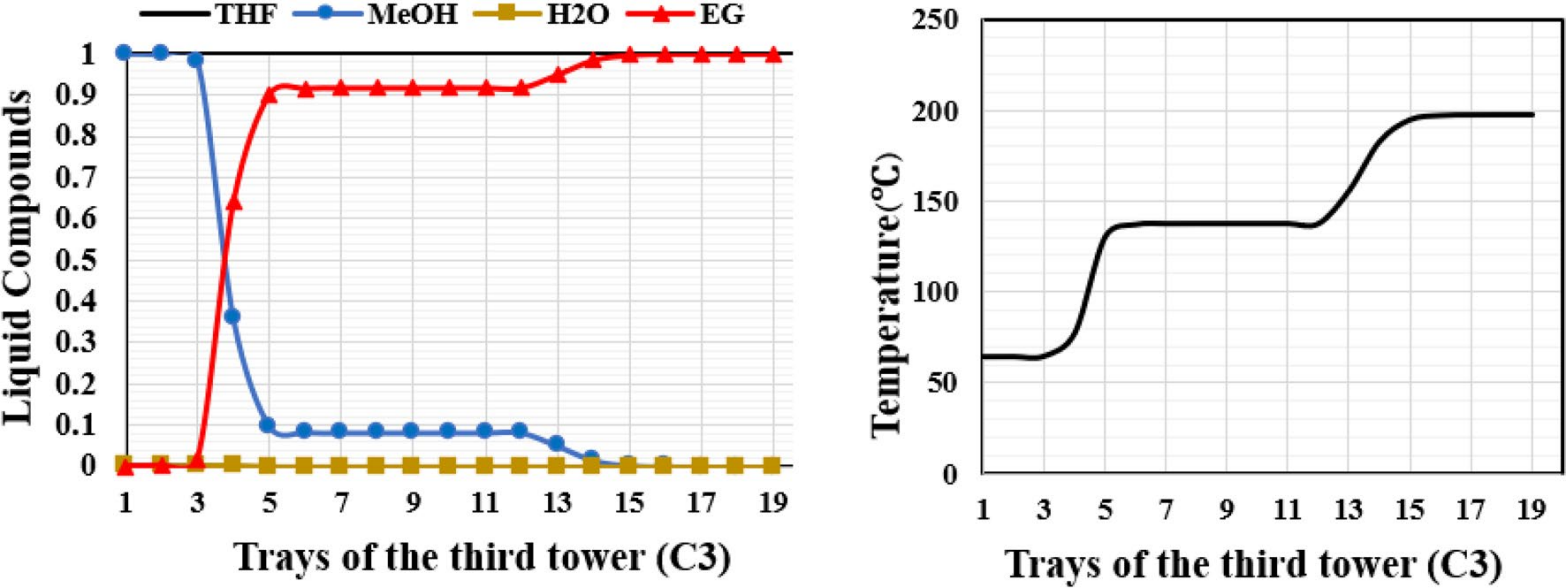
Temperature and composition profiles for OD column of the new design (II).

Figures 19 and 20 illustrate the composition and temperature profiles on the column trays in the liquid phase for each of the columns of the new design (II).

The RD column of the new design (II) exhibits temperature and concentration profiles that are comparable to those of the new design (I). Due to differences in solubility, methanol dissolves in the EG solvent upon the addition of EG feed on tray 3, as illustrated in Fig. 19, and nearly pure THF exits the top of the column. The temperature profile of this column shows that as the solvent is added, the temperature rises up to the feed entrance point and then falls after that point. At the bottom of the column, the temperature increases due to the increase in the concentration of ethylene glycol.

In the third column of this design, EG solvent is separated from methanol by differences in their boiling points. Methanol and EG leave the column as the top and bottom products, respectively. As depicted in Fig. 20, the temperature profile of this column remains relatively uniform from tray 12 to tray 5. With the addition of reflux from the top, the purity of methanol increases, and the temperature decreases. At the bottom of the column, where the solvent purity is high due to the high boiling point of this substance, the temperature increases to 197.2 degrees Celsius.

### Economic analysis

**Figure 21:**
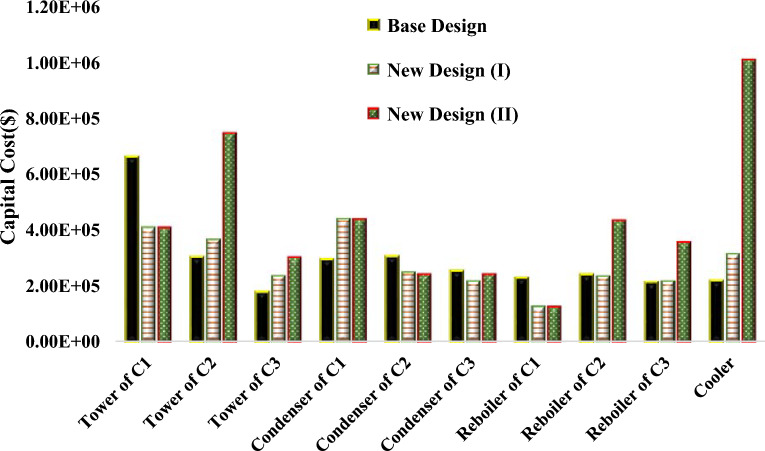
Capital costs of the main equipment items in the base and new designs.

Some design characteristics of the main equipment items available in the new designs and the base design processes are summarized in Table 5. Also, the capital costs of the equipment items have been compared in Fig. 21. Table 5 presents the design parameters for each process separately. The costs of energy, EO feed and the solvents are presented in Table 6. Also, in Table 7, economic features of the new designs can be compared to those of the base design.

**Table 5:** Main design characteristics of the main equipment items.

Equipment	Variables	Base design (extractive distillation)	New design (I) (reactive extractive distillation with solvent DMSO)	New design (II) (reactive extractive distillation with solvent EG)
C1	${{\text{N}}}_{{\text{T}}1}$	61	31	31
	${{\text{ID}}}_{1},{\text{m}}$	1.765	1.92	1.92
	${{\text{Q}}}_{{\text{C}}1},{\text{MW}}$	3.527	6.481	6.481
	${{\text{Q}}}_{{\text{R}}1},{\text{MW}}$	3.976	1.581	1.581
C2	${{\text{N}}}_{{\text{T}}2}$	34	32	50
	${{\text{ID}}}_{2},{\text{m}}$	1.348	1.684	2.31
	${{\text{Q}}}_{{\text{C}}2},{\text{MW}}$	3.72	2.695	2.59
	${{\text{Q}}}_{{\text{R}}2},{\text{MW}}$	4.309	4.113	10.62
C3	${{\text{N}}}_{{\text{T}}3}$	16	21	19
	${{\text{ID}}}_{3},{\text{m}}$	1.527	1.569	2.17
	${{\text{Q}}}_{{\text{C}}3},{\text{MW}}$	2.799	2.177	2.43
	${{\text{Q}}}_{{\text{R}}3},{\text{MW}}$	3.57	3.625	7.86
Cooler	${{\text{Q}}}_{{\text{Cooler}}},{\text{MW}}$	1.65	2.85	13.44
Makeup	m, kg/h	7.8	0.7	1.67
EO	m, kg/h	-	8259.97	8259.97

**Table 6:** Comparison of base design and two new designs in terms of total energy consumption, required raw materials (feedstock + makeup), and total operational cost.

Design	TOC ($/yr*)*			
	Total energy cost	Cost of raw materials		
		EO (feed)	EG (makeup)	DMSO (makeup)
Base design	$$27.81\times {10}^{5}$$	0	0	$$2.9\times {10}^{5}$$
Design (I)	$$22.9\times {10}^{5}$$	$$6.64\times {10}^{7}$$	0	$$2.6\times {10}^{4}$$
Design (II)	$$49.8\times {10}^{5}$$	$$6.64\times {10}^{7}$$	$$1.14\times {10}^{4}$$	0

**Table 7:** The economic features of the base and new designs.

Economic indicators	Base design (extractive distillation)	New design (I) (reactive extractive distillation with solvent DMSO)	New design (II) (reactive extractive distillation with solvent EG)
TOC, $/year	3.07E+06	6.87E+07	7.13E+07
TCC, $	2.91E+06	2.86E+06	4.31E+06
TAC, $/year	4.04E+06	6.97E+07	7.28E+07
TAC-EG $/year	4.04E+06	− 9.80E+06	− 6.7E+06

In terms of total operational cost, the base design has a lower value compared to the new designs (I) and (II). This can be justified by considering the amount of energy consumption in the reboilers, the cost of ethylene oxide feedstock, and makeup solvent. In fact, since the separation power of the EG solvent is less than that of DMSO solvent, a greater amount of EG solvent is used to achieve the desired product purity. This leads to an increase in the operational cost related to the heat load of the reboilers in the new design (II). Furthermore, the operational cost related to the ethylene oxide feedstock, which is used for the reaction in the new designs, has caused the operational costs to be significantly higher compared to the base design. The operational cost related to makeup to compensate for the loss of solvent in the base design is also higher compared to the two new designs.

The total capital cost in the new design (I) is lower compared to the base and new design (II). According to Fig. 21, in the new design (I), by adding the reaction to the first column and removing water from the azeotropic system, separation is achieved with fewer trays, which has led to a reduction in the total capital cost of the new design (I) compared to the base design. In the new design (II), due to the lower separation power of ethylene glycol solvent, the size of the related equipment has also increased. Ultimately, the total capital cost in the new design (II) is higher compared to the other two designs.

In overall, considering the total operational and capital costs of the designs, the total annual cost (TAC) was calculated. The base design has a lower TAC (4.04 million dollars) compared to the proposed new designs. The reason for the higher TAC in the proposed new designs is related to the higher cost of purchasing ethylene oxide feedstock for the reaction in the first column. However, it should be noted that in the new designs, in addition to producing methanol and THF, a valuable by-product of ethylene glycol is produced at a rate of 11,635 kg/h. Given the price range of this product (0.8–0.9 US$/kg ), on average, it is possible to generate revenue of 79.5 million dollars per year from the sale of ethylene glycol in these designs. Therefore, in the new designs, although the total annual cost is higher compared to the base design, the revenue generated from the sale of EG can compensate for the TAC in less than one year, resulting in revenues of 9.8 and 6.7 million dollars for the new designs (I) and (II) respectively, in the same year.

It should be noted that economic calculations are always influenced by fluctuations in the prices of raw materials and products. Therefore, the economic results obtained for the superiority of Model 1 over the base design significantly depend on the price of ethylene oxide. The increase in the price of this compound affects operational costs, and economic calculations indicate that assuming a constant price for ethylene glycol, a fifteen percent increase in the price of ethylene oxide would result in almost zero net income from the annual sales of ethylene glycol in Model 1. However, it should be considered that ethylene glycol is obtained from the hydrolysis of ethylene oxide, and an increase in the price of ethylene oxide can lead to an increase in the price of ethylene glycol as well.

### Environmental indicators

**Figure 22:**
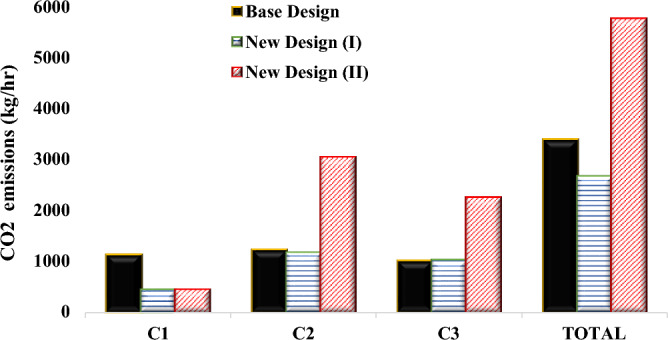
The carbon dioxide emissions of the new designs and the base designs.

Referring to the heat duties of the reboilers outlined in Table 5, the calculation of carbon dioxide emissions was performed utilizing Eq. (12). The quantities of carbon dioxide generated in the distillation columns for the base design, new design (I) and new design (II) are depicted in Fig. 22. As seen in this Figure, the lowest amount of carbon dioxide production was in the first column of the new designs, and it is 21.60% less compared to the first column of the base design. The exothermic reaction between ethylene oxide and water is the cause of this reduction, as it eliminates the water and, as a result, lowers the reflux requirement of this column for separating the remaining components by 29.47% when compared to the first column of the base design. This lowers the heat load of the reboiler in this column.

Because of the large amount of recycled EG solvent in the second and third columns of the new design (II) and the resulting significant rise in the heat load of the reboilers, a notable amount of carbon dioxide is produced. As a result, the new design (I) with a total production of 2673.2 kg/h carbon dioxide produces 21.39% and 53.63% less carbon dioxide compared to the base design and the new design (II), respectively. So the new design (I) has an environmental advantage.

## Conclusion

Two new processes for the separation of a ternary azeotropic mixture of water, methanol, and THF based on reactive/extractive distillation systems were proposed and simulated in this work. The innovation in this research involves the use of reactive distillation to remove water from the azotropic mixture through the reaction of water and ethylene oxide in the first distillation column. This simplifies the separation process, and reduces the number of column trays. Additionally, two solvents, dimethyl sulfoxide and ethylene glycol, were examined in the extractive distillation section. The extractive solvents in the novel designs (I) and (II) were dimethyl sulfoxide and ethylene glycol, respectively. Subsequently, these designs were compared economically and environmentally with a conventional extractive distillation process (the base design). The new design (I) has a lower total capital investment cost than the base design and the new design (II). Because it does not use ethylene oxide feed, the base design has lower operating costs than the new designs. However, the proposed new designs, by selling the ethylene glycol product, not only offset this cost increase but also produce a significant profit within one year.The new designs (I) and (II) generate annual incomes of 9.8 million dollars and 6.7 million dollars, respectively. The new design (I) generates a total carbon dioxide output of 2673.18 kg/h, resulting in a 21.39% reduction compared to the base design and a 53.63% decrease compared to the new design (II) from an environmental standpoint. A summary of some further findings from this study is provided below.

- Water removal from the ternary azeotropic mixture of water/methanol/THF aids separation because water is removed from the azeotropic system of water-THF, which has a minimum boiling azeotrope.
- Ethylene glycol solvent has less capability to dissolve methanol compared to dimethyl sulfoxide solvent, which results in the consumption of a larger amount of ethylene glycol solvent in the extractive distillation stage compared to dimethyl sulfoxide solvent, leading to larger equipment size and operational costs.
- Overall, in terms of environmental and economic factors, the new design (I) outperformed the base design and the new design (II), and it was chosen as the best process to replace the conventional extractive distillation method.

### Supplementary Information

Supplementary Table 1.

## Data Availability

The data used in this study were generated through simulation and would be available from the corresponding author on reasonable request.

## List of symbols

ED: Extractive distillation
RED: Reactive extractive distillation
DMSO: Dimethyl sulfoxide
EO: Ethylene oxide
THF: Tetrahydrofuran
EG: Ethylene glycol
MEOH: Methanol
ε: Material price, $/kg
DEHI: Double effect heat integration
TAC: Total annual cost, $/yr
TOC: Total operating cost, $/yr
TCC: Total capital cost, $
$${{\text{TAC}}}_{{\text{RD}}}$$: Total annual cost of reaction distillation tower ,$/yr
$${{\text{TOC}}}_{{\text{RD}}}$$: Total operating cost of reaction distillation tower, $/yr
$${{\text{TCC}}}_{{\text{RD}}}$$: Total capital cost of reaction distillation tower, $
VRC: Vapor recompression
HX: Heat exchanger
NT: Total number of trays
Q: Heat duty of heat exchanger, kW
U: Heat transfer coefficient, kW/m^2^ K
∆T: Temperature difference, K
$${{\text{D}}}_{i}$$: Distillate rates of column i, kmol/h
$${{\text{RR}}}_{i}$$: Reflux ratio of column i
$${{\text{B}}}_{i}$$: Bottom rates of column i, kmol/h
$${{\text{Q}}}_{{\text{fule}}}$$: Heat released by fuel, kJ/h
NHV: Net heating value, kJ/kg
$${{\text{C}}}_{i}$$: Column i
r: Reaction rate, kmol/m^3^ s
m: Mass flow, kg/h
$${{\text{Q}}}_{{\text{Ci}}}$$: Heat duty condenser of column i, MW
$${{\text{Q}}}_{{\text{Ri}}}$$: Heat duty reboiler of column i, MW
$${{\text{Q}}}_{{\text{R}}\left({\text{RD}}\right)}$$: Heat duty reboiler of reaction distillation tower, MW
$${{\text{Q}}}_{{\text{C}}\left({\text{RD}}\right)}$$: Heat duty condenser of reaction distillation tower, MW
$${{\text{ID}}}_{(RD)}$$: Diameter of reactive distillation tower, m
$${{\text{ID}}}_{i}$$: Diameter of column i, m
$${{\text{N}}}_{{\text{Ti}}}$$: Total number of trays of column i
$${{\text{N}}}_{{\text{T}}({\text{RD}})}$$: Total number of trays of reaction distillation tower
$$\alpha $$: Ratio of molecular weight between CO_2_ and C
P: Pressure, atm
X: Mole fraction
A: Area, m
L: Length, m
T: Temperature, °C
$$\mathfrak{J}$$: The cost of steam consumption, $/GJ
NRTL: Nonrandom-two-liquid
$${{\text{A}}}_{{\text{R}}\left({\text{RD}}\right)}$$: The heat transfer surface reboiler of reaction distillation tower, m^2^
$${{\text{A}}}_{{\text{C}}({\text{RD}})}$$: The heat transfer surface of condenser of reaction distillation tower, m^2^
