# Supplementary material for: A novel reactive-extractive distillation process for separation of water/methanol/tetrahydrofuran mixtures
Source: Sci Rep. 2024 Jan 22;14:1931. doi: 10.1038/s41598-024-52427-3 (PMC10803736; doi:10.1038/s41598-024-52427-3)
Supplement: Supplementary file 1 — Supplementary Table 1. [file 41598_2024_52427_MOESM1_ESM.docx]

**Appendix**

**Table A- Binary interaction parameters of the NRTL activity model.**

| **Component i** | **Component j** | **AIJ** | **AJI** | **BIJ** | **BJI** | **CIJ** |
| --- | --- | --- | --- | --- | --- | --- |
| THF | MEOH | 0.000 | 0.000 | 224.151 | 63.105 | 0.300 |
| THF | H2O | 1.214 | 4.760 | 157.781 | -733.402 | 0.473 |
| THF | EG | -1.755 | 0.682 | 1083.195 | 32.862 | 0.400 |
| THF | DMSO | 0.000 | 0.000 | 347.549 | 74.937 | 0.300 |
| MEOH | H2O | -0.693 | 2.732 | 172.987 | -617.269 | 0.300 |
| MEOH | EG | 33.330 | 0.175 | -10000.000 | -322.924 | 0.300 |
| MEOH | DMSO | 0.000 | 0.000 | 30.597 | -331.156 | 0.300 |
| H2O | EO | 0.000 | 0.000 | 188.688 | 868.971 | 0.300 |
| H2O | EG | 0.348 | -0.057 | 34.823 | -147.137 | 0.300 |
| H2O | DMSO | -1.245 | 1.752 | 586.801 | -1130.216 | 0.300 |
| EG | DMSO | 0.000 | 0.000 | -407.991 | 125.280 | 0.300 |
| THF | EO | 0.000 | 0.000 | -99.311 | 272.463 | 0.300 |
| MEOH | EO | 0.000 | 0.000 | 61.017 | -24.941 | 0.300 |
| EO | EG | 0.000 | 0.000 | 157.545 | -142.372 | 0.300 |
| EO | DMSO | 0.000 | 0.000 | -332.399 | 426.424 | 0.300 |
